# Supplementary material for: Application of multivariate time-series model for high performance computing (HPC) fault prediction
Source: PLoS One. 2023 Oct 17;18(10):e0281519. doi: 10.1371/journal.pone.0281519 (PMC10581458; doi:10.1371/journal.pone.0281519)
Supplement: S1 Appendix — (DOCX) [file pone.0281519.s001.docx]

**Appendix A**

Symbol table：

| Abbreviation | Full name |
| --- | --- |
| HPC | High Performance Computing |
| HDBSCAN | Hierarchical Density-Based Spatial Clustering of Applications with Noise |
| DBSCAN | Density-Based Spatial Clustering Algorithm with Noise |
| DevOps | Development Operations |
| AIOps | Artificial Intelligence for IT Operations |
| CNN | Convolutional Neural Network |
| BiLSTM | Bi-directional Long Short-Term Memory |
| MAE | Mean Absolute Error |
| RMSE | Root Mean Square Error |
| SVR | Support Vector Regression |
| XGboost | Extreme Gradient Boosting |
